# Psychological interventions for cancer-related post-traumatic stress disorder: narrative review

**DOI:** 10.1192/bjb.2023.42

**Published:** 2024-04

**Authors:** Daniel Anderson, Victoria Jones

**Affiliations:** 1 The Christie NHS Foundation Trust, Manchester, UK; 2 Greater Manchester Mental Health NHS Foundation Trust, Manchester, UK

**Keywords:** Cancer, psychological intervention, individual psychotherapy, post-traumatic stress disorder, liaison psychiatry

## Abstract

**Aims and method:**

This narrative review updates the evidence base for cancer-related post-traumatic stress disorder (PTSD). Databases were searched in December 2021, and included EMBASE, Medline, PsycINFO and PubMed. Adults diagnosed with cancer who had symptoms of PTSD were included.

**Results:**

The initial search identified 182 records, and 11 studies were included in the final review. Psychological interventions were varied, and cognitive–behavioural therapy and eye movement desensitisation and reprocessing were perceived to be most efficacious. The studies were also independently rated for methodological quality, which was found to be hugely variable.

**Clinical implications:**

There remains a lack of high-quality intervention studies for PTSD in cancer, and there is a wide range of approaches to managing these conditions, with a large heterogeneity in the cancer populations examined and methodologies used. Specific studies designed with patient and public engagement and that tailor the PTSD intervention to particular cancer populations under investigation are required.

Psycho-oncologic trauma is defined as a deeply distressing or disturbing experience related to the experience of being diagnosed with, or treated for, cancer,^1^ and is a risk factor for developing post-traumatic stress disorder (PTSD).^2^ Cancer-related PTSD is defined as patients who become re-traumatised by here-and-now experiences (such as having cancer) and/or through evoking past traumatic experiences (typically relating to adverse issues pertaining to social deprivation, comorbidities and psychologic trauma from neglect and abuse). There is evidence that cancer-related outcomes may be negatively associated with pre-cancer diagnosis PTSD as a result of adverse childhood experiences and adverse adulthood experiences^3^ (see Felitti et al^4^ for their definition and measurement). Prevalence estimates of cancer-related PTSD range between 7 and 14%,^1^ with an additional 10–20% of patients experiencing subsyndromal post-traumatic stress symptoms.^5,6^ There is a developing evidence base in understanding trauma experiences among people living with cancer, and the effect these trauma experiences have on health and well-being outcomes.^7,8^ Consequently, mental health services for patients with cancer are urged to accommodate the needs for patients with current and/or past traumatic experiences.^9^

PTSD is known to reduce quality of life in people living with cancer^10^ and concordance with cancer care, such as drug adherence; this negatively effects morbidity and mortality.^11,12^ There is considerable diagnostic heterogeneity and diagnostic overlap in this group of patients, who may present to cancer services with pre-existing PTSD, and those that develop *de novo* PTSD as a result of their cancer experience.^13^ Recent authors have commented on the alteration of criteria in the DSM-5, and argue that cancer may not necessarily constitute a traumatic event if no sudden or catastrophic events occur.^14,15^ It has also been suggested that careful consideration ought to be paid to distinguishing between PTSD and adjustment disorder, anxiety and depression, and that cancer-related PTSD remains a complex diagnostic issue.^15^ This diagnostic ambiguity is relevant given the complexity of mental health problems in patients that is further compounded by risk factors for pre-existing PTSD, which include deprivation, adverse childhood experiences and adverse adulthood experiences.^16^ (The DSM-5 criteria for PTSD are: stressor, intrusion symptoms, avoidance, negative alterations in cognitions or mood, alterations in arousal and reactivity, duration and functional significance.^17^)

PTSD is known to be prevalent (14%) in the oncology population, alongside other psychiatric comorbidities such as depression (16.3%), adjustment disorder (19.4%) and anxiety (10.3%).^18^ Although concordance with cancer treatment is important, it is also known that when patients have mental health problems, their physical health outcomes in general are worse.^19–21^ This narrative review will update the evidence base for psychological interventions in patients living with cancer-related PTSD and part of addressing these needs in the psychiatric population.

## Method

This narrative review identified the evidence base of existing and applicable psychological approaches to cancer-related PTSD that can translate across to people living with cancer (Quality Improvement and Clinical Audit Committee 3055).

### Narrative literature review approach

A narrative literature review aims to answer a specific clinical question by using defined criteria for the literature search. The results of the search are then reviewed and critiqued for the quality of the evidence. The question posed was: ‘What are the evidence-based psychological approaches to PTSD in people living with cancer?’. To answer the question, the PICO method was used, which addresses the population of interest (P), the intervention to be considered (I), the relevant control population or intervention (C) and the outcomes (O).^22^ The PICO method was therefore formulated with P as patients with cancer and post-traumatic stress symptoms (PTSD), I as psychological approaches to PTSD, C as patients with cancer without PTSD and O as improvement in PTSD symptoms and/or engagement into cancer care.

PTSD as the primary outcome was defined with the criteria determined by specific psychometric tools: Structured Clinical Interview for DSM-IV or -5 (SCID),^17^ Clinician-Administered PTSD Scale (CAPS),^23^ Post-Traumatic Diagnostic Scale (PDS),^24^ Short Post-Traumatic Disorder Rating Interview (SPRINT),^25,26^ the Impact of Events Scale (IES)^26^ or revised Impact of Events Scale (IES-R),^27^ and the PTSD Checklist (both the civilian version and revised versions) (PCL-C).^28^ By doing so, this updated narrative review is consistent with the psychometric tools used in the two previous reviews.^15,29^

### Search strategy

Each database was searched for keywords and results were added to the synonym column (see Table 1).

**Table 1:** Search strategy

Main keywords	Synonyms
Post-traumatic stress disorder	Traumatic, trauma, PTSD, cancer-related distress
Psychological approach	Psychotherapy, counselling, psychological techniques, psychodynamic psychotherapy, cognitive–behavioural therapy (CBT), narrative therapy, eye movement desensitisation and reprocessing (EMDR), interpersonal therapy
Cancer	Tumour, metastatic, cancer, neoplasm, oncology
Improvement	Engagement, reduction, effect, observation

### Inclusion and exclusion criteria

Papers were included if the following criteria were met: the study was published in a peer-reviewed journal; the entire paper published in the English language; it was a primary research paper; the study was related to cancer; the study addressed the relationship between cancer and PTSD, using the validated psychometric tools from the two previous reviews; and studies using mixed tools to measure multiple psychiatric diagnoses were included if the outcome data presented those scoring highly specifically for PTSD.

Studies that did not meet the above criteria were excluded after inspecting the title and abstract. For those studies that were included, entire papers were obtained and assessed for eligibility. There was a preference for papers published in the past 20 years, to provide the most relevant evidence-based for current cancer care.

### Databases searched

A narrative review of healthcare databases was conducted in December 2021. The following electronic databases were subsequently searched: Medline (general medical database), EMBASE (Excerpta Medica Database), CINAHL (Cumulative Index to Nursing and Allied Health Literature), PsycINFO (Psychology and Allied Fields), BNI (British Nursing Index) and PubMed (general medical database). Medical Subject Headings (MeSH), keywords and synonyms used are detailed in Table 1. Each paper was reviewed for usefulness, and general review papers, single-case studies and non-intervention papers were excluded. Papers that had been included in previous reviews were included as part of previous narrative and systematic review papers, but were not separately considered so as to avoid duplication and were therefore excluded from the search.

### Data extraction

An analytical framework was created to extract relevant themes. Data was recorded on standardised forms that checked eligibility, recorded the details of the paper and extracted the main themes. The two authors (D.A. and V.J.) independently performed the data extraction, using a standardised form. The results were organised by author, year of publication, objective, study design and results. Papers were then critically appraised with a standardised method (Critical Appraisal Skills Programme checklists).^30^ This tool has been validated to ensure a standardised rigour to the approach. The following variables were extracted and used to focus each review to the specific aim of the study:

- general: author, year, title, journal, country, total number of participants and study design;
- participants: gender, age range, cancer type and PTSD psychometric tool used in the study;
- intervention: type of intervention, frequency, delivery mode (face to face, telephone, group or individual), randomisation method if discussed, drop-out data;
- results: main outcome measures, narrative findings and effect sizes if available.

The risk of bias was examined for each paper. National Institutes of Health quality assessment tools were used for controlled intervention studies, randomised controlled trials (RCTs), case series and studies with no control group. One narrative review had already performed a quality assessment with such tools and was therefore not repeated.^15^ A meta-analysis was not possible because of the clinical variability of the interventions and outcomes measures, as well as methodological variability. Measures used include the PTSD Symptom Scale, SCID (for the DSM-IV), PCL, IES, SPRINT, CAPS, PDS and Posttraumatic Stress Syndrome Scale. Of the individual studies, two commented on the cut-off score used,^31,32^ whereas others did not comment on cut-off scores at all, rather discussing the comparison in scores pre- and post-intervention.

## Results

**Fig. 1:**
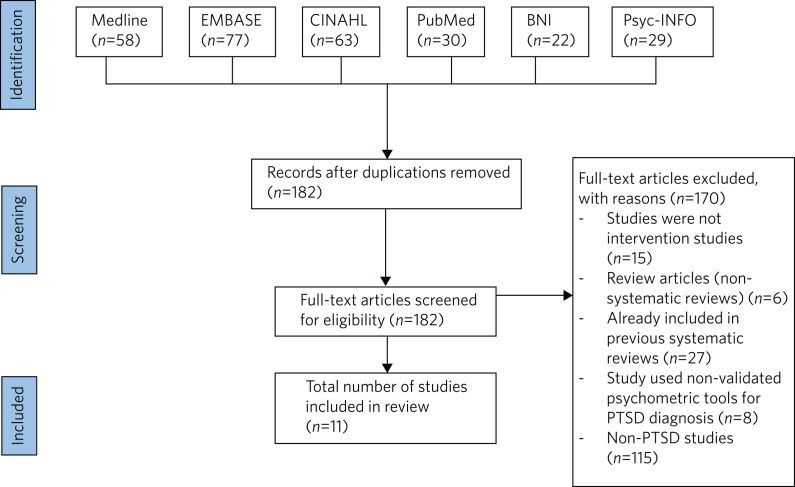
Preferred Reporting Items for Systematic Reviews and Meta-Analyses (PRISMA) summary. The following electronic databases were searched: Medline (general medical database), EMBASE (Excerpta Medica Database), CINAHL (Cumulative Index to Nursing and Allied Health Literature), PsycINFO (Psychology and Allied Fields), BNI (British Nursing Index) and PubMed (general medical database). PTSD, post-traumatic stress disorder.

Table 2 and Fig. 1 detail the results of the narrative review, and Table 3 shows the quality assessment of the studies.

**Table 2:** Summary of included papers

Author	Study location	Study characteristics	Intervention and setting	Control	PTSD measure	Narrative findings
Individual studies						
Chu et al^39^	USA	Pre- and post-study with no control, *n* = 136, 100% women, mean age 57.8 years, breast cancer	Expressive writing intervention using cognitive reappraisal and emotional disclosure. Group-based interventions over 3 weeks. Three types of writing groups were examined: ‘cancer-fact’, self-regulation and enhanced self-regulation	None beyond comparing the three interventions to each other	PSS	Significant differences in PTSD symptoms for all groups. No significant differences in follow-up at 1, 3 and 6 months. Drop-out data not described
Farahimanesh et al^36^	Iran	Pre- and post-study with no control; *n* = 60; 68.3% women; mean age 49 years; breast, colon, lung and bladder cancers	Two approaches compared: COMET (seven sessions done weekly and individually) versus MEST (six sessions done weekly and individually). COMET focuses on altering dysfunctional self-representations. MEST targets autobiographical memory deficits. Both interventions are a combination of CBT and psychoeducation	None beyond comparing the two interventions to each other	PCL SCID	COMET group had significantly fewer PTSD symptoms compared with the MEST group at both immediate post-intervention and at 3-month follow-up. Drop-out data not described
Hamidian et al^35^	Iran	RCT, *n* = 85, 100% women, mean age 43 years in intervention and 40.3 years in control, breast cancer	Group cognitive–emotional training (five sessions delivered twice weekly) versus treatment as usual	Treatment as usual	PCL	Mean PTSD score in post-test and mean changes in stress score of intervention group were significantly less than those in control group. Similar pre-study PTSD scores in both groups. Simple envelope method used for randomisation. Drop-out data not described
Monti et al^34^	USA	Pre- and post-study with no control; *n* = 7; 100% women; mean age 51 years; Hodgkin's lymphoma, breast and cervix cancers	‘Neuro-emotional technique’ using desensitisation and complimentary therapy (three sessions, frequency unknown)	None	IES	71% improvement on IES score was statistically significant. Effect size of 0.5 on IES. Drop-out data was not described
Offidani et al^37^	USA	Pre- and post-study with no control, *n* = 64, 100% women, mean age 60 years, breast cancer	4-week programme of meditation aimed at improving attention and awareness while building essential skills to deal with stress. Eight 90-minute group sessions over 4 weeks followed by a cognitive–behavioural learning programme	None	IES	Significant decrease in IES scores for family and job stress, but not for money or health stress. Drop-out rate was 33/64
Ochoa et al^38^	Spain	Case–control study; *n* = 126; 100% women; mean age 48.9 years; breast, uterus, Hodgkin's lymphoma, non-Hodgkin's lymphoma, colon, leukaemia, ovary and rectum cancers	Tested a positive psychotherapy technique (PPC). 12 weekly sessions of 90–120 min duration, focusing on assimilation of cancer experience and encouraging accommodation and personal transformation (growth) from illness experience. Intervention used a manualised programme and guide	Waiting list	PCL	In the PPC group, there were significant differences from the control. Differences included a decrease in distress, a decrease in PTSD symptoms and an increase in post-traumatic growth. Differences were maintained at 3 and 12 months. Drop-out data: control group rate of 10/53, intervention group rate of 29/73
Roberts^32^	USA	Pre- and post-study with no control, *n* = 35, 77% women, mean age 64 years, ‘various types of cancer in different stages’	Group-based EMDR (one 90 min EMDR session on two consecutive days)	Intervention compared with 1- month delayed treatment group	SPRINT	Significant differences in PTSD symptoms: 54% of pre-test scores on SPRINT were above a cut-off score of 14, 29% remained above cut-off post-test and 26% remained above cut-off at 1-month follow-up. Drop-out rate: 1/35
Rissanen et al^31^	Sweden	RCT, *n* = 425, 100% women, mean age 59 years, breast cancer	Intervention described as a stepped-care stress management, educationally based approach along with CBT techniques. Group intervention compared with individual treatment versus a one-off education event	Patients deemed non-stressed after education event were used as the control	IES	No significant differences between groups for the educational event. For both individual and group interventions, there were significant improvements. There were no differences between individual and group interventions, but individual therapy was preferred. Drop-out data: 54% completed the group intervention and 91% completed the group intervention
Sarizadeh et al^33^	Iran	RCT, *n* = 52, 100% women, mean age range for two groups: 43.3–53.2 years, breast cancer	Acceptance and commitment therapy. 8 weeks of weekly sessions, including a patient group and a survivors’ group	Same two groups for controls – controls offered four sessions of treatment after completing the study	PCL	Significant improvement in PCL scores for patient and survivor groups post-intervention. Drop-out data not described
Reviews						
Dimitrov et al^15^	Italy, Mexico, USA and Australia	Narrative review of eight international papers: four RCTs, one pre- and post-test study and three case study/case series. Total *n* = 670, 88% women, age range: 40–58.6 years, multiple common cancer diagnoses across all disease groups included	Interventions included group and individual EMDR, telephone and face-to-face CBT, individual and group counselling/psychotherapy, complementary therapies, stress management and psychoeducation	Five out of eight studies used a control group	SCID CAPS IES PDS PSS SPRINT PCL	Individual EMDR versus CBT (weekly for 8 weeks): EMDR had the best outcomes. CBT versus counselling (six sessions over 6 weeks, with a booster session at 10 weeks): CBT had the best outcomes. Telephone counselling and resource booklet (16 sessions over 12 months) versus booklet alone: counselling had the best outcomes. Complimentary therapy (twice weekly for 12 months) versus a standard support group (weekly for 12 weeks): support group had the best outcomes
Nenova et al^29^	Countries not described. Predominantly White populations	Review of RCTs using CBT for cancer-related PTSD; 19 studies in total; total *n* = 3272; 16 studies had either 99% or 100% women, and one study had 100% men; age range: 42–64 years; various cancer diagnoses, including breast (predominantly), ‘gynaecological cancer’, prostate and gastrointestinal	Range of interventions, including groups (four studies), couples (one study) and individual formats (14 studies). Number of sessions varied from two to ten over a year	All included studies were RCTs; controls included waiting list, assessment only, treatment as usual or an educational event	IES PTSS PCL CAPS	Six trials showed a significant effect on post-traumatic stress symptoms. Effect size analysis showed intervention effects were small and ranged from –0.138 to 0.006 for intrusion, and from −0.025 to 0.048 for avoidance. Effect sizes indicated interventions with CBT components did not have a statistically significant effect on either intrusion or avoidance scores. There was insufficient evidence to make a firm conclusion about CBT and cancer-related PTSD

PTSD, post-traumatic stress disorder; PSS, PTSD Symptom Scale; COMET, competitive memory training; MEST, memory specificity training; CBT, cognitive–behavioural therapy; PCL, PTSD checklist; SCID, Structured Clinical Interview for DSM-IV or -5; RCT, randomised controlled trial; IES, Impact of Event Scale (original or revised); PPT, positive psychotherapy technique; EMDR, eye movement desensitisation and reprocessing; SPRINT, Short Post-Traumatic Disorder Rating Interview; CAPS, Clinician-Administered PTSD Scale; PDS, Post-Traumatic Diagnostic Scale; PTSS, Posttraumatic Stress Syndrome Scale.

**Table 3:** Quality review

Study	Study type	Quality tool	Quality rating
Dimitrov et al^15^	Narrative review	NIH quality assessment tool	Good
Nenova et al^29^	Systematic review	NIH quality assessment tool	Fair
Monti et al^34^	Pre–post study with no control	NIH quality assessment tool	Poor
Roberts^32^	Pre–post study with no control	NIH quality assessment tool	Fair
Hamidian et al^35^	Randomised controlled trial	NIH quality assessment tool	Good
Farahimanesh et al^36^	Pre–post study with no control	NIH quality assessment tool	Fair
Rissanen et al^31^	Randomised controlled trial	NIH quality assessment tool	Fair
Offidani et al^37^	Pre–post study with no control	NIH quality assessment tool	Poor
Ochoa et al^38^	Case–control study	NIH quality assessment tool	Fair
Sarizadeh et al^33^	Randomised controlled trial	NIH quality assessment tool	Poor
Chu et al^39^	Pre–post study with no control	NIH quality assessment tool	Poor

NIH, National Institutes of Health.

Of the 11 included papers, the studies broadly fell into three distinct groups: predominantly cognitive–behavioural therapy (CBT)-based studies, predominantly eye movement desensitisation and reprocessing (EMDR)-based studies and other modalities. Two studies provided an overview of treatment in cancer-related PTSD, through narrative review^15^ and a review of RCTs.^29^ The narrative review examined eight studies that address EMDR, CBT, complimentary therapies and counselling over a range of formats, such as individual, group, self-management and telephone therapy. The review of RCTs examined 19 studies that used CBT in various formats (individual, group and couples); 68% of these studies were found to not be helpful in cancer-related PTSD. Although there was significant heterogeneity between the studies, individual EMDR seemed to have the best evidence when trialled head-to-head with CBT.

One study used a CBT approach,^31^ one study used EMDR^32^ and one study focused on acceptance and commitment therapy.^33^ Finally, six studies used novel approaches, including neuro-emotional techniques, cognitive–emotional training, competitive memory training, meditation, ‘positive psychotherapy techniques’ and expressive writing.^34–39^ There were noticeable differences in type of intervention, participant characteristics and outcome assessments across the studies. Duration of intervention also differed, ranging from 3 weeks to 22 weeks, and post-intervention follow-up varied from 90 days up until 12 months.

There was a large variability in types and stages of cancer studied, ranging from haematological malignancies to oral cancer, and some studies focused on one cancer type only, whereas others included participants with a range of different cancers. There was also considerable variability in patient demographics, including gender, age and social circumstances. These factors contribute to notable heterogeneity in this narrative review.

### CBT and derivative approaches

An outcome of the narrative review by Dimitrov et al^15^ examined CBT versus other modalities, including EMDR (both individual and group), counselling, complimentary therapy and psychoeducation. Telephone CBT was compared with a resource workbook done over ten sessions across 10–16 weeks). No control group was included; however, it seemed that telephone CBT provided the best outcome. CBT was also compared with counselling (six sessions over 6 weeks, with a booster session at 10 weeks), with CBT resulting in the best improvement for PTSD symptoms.

Nenova et al^29^ reviewed several randomised controlled trials that used CBT for cancer-related PTSD (19 studies in total). A range of interventions were examined that used group, couples and individual formats. Additional techniques to modify the CBT included mindfulness, psychoeducation, self-management and anxiety management. Control groups varied across the included studies, including wait-list controls, day-long seminars, treatment as usual, a psychological assessment only, an unstructured psychoeducational support group, supportive counselling, resource directory booklet provision, unstructured supportive counselling, medical information education (no psychological intervention) and coping skills training. In terms of effectiveness, 68% of studies found no an effect on cancer-related traumatic stress symptoms. Four studies found intervention participants experienced reductions in intrusion, avoidance and hyperarousal symptoms compared with controls. One study found a reduction in intrusion and avoidance, but not numbing or hyperarousal; however, participants were less likely to meet the diagnostic criteria for PTSD at 12-month follow-up in the intervention group.

One study found that a CBT-based support group led to greater reductions in re-experiencing symptoms and arousal, but not in avoidance, when compared with complementary and alternative medicine intervention. Overall effect size analysis showed intervention effects were small and ranged from −0.138 to 0.006 for intrusion, and from −0.025 to 0.048 for avoidance. Effect sizes indicated interventions with CBT components did not have a statistically significant effect on either intrusion or avoidance scores.

There were three studies included in this review that used CBT-derivative approaches to modify the original CBT technique. Hamidian et al^35^ adopted a CBT-derivative approach using so-called group cognitive–emotional training (five sessions delivered twice weekly) compared with treatment as usual, in a cohort of 85 participants. The mean PTSD score in post-test and the mean changes in stress score of the intervention group were significantly less than those in control group. Pre-study PTSD scores were similar in both groups. Farahimanesh et al^36^ compared so-called competitive memory training (seven sessions delivered weekly) and memory specificity training (six sessions delivered weekly). Both interventions appeared to be a combination of CBT and psychoeducation, with the first group more cognitively focused on self-perceptions and the second group focusing on recall of memories. It was not clear within the paper what the interventions used as a psychological approach, and there were no control groups for comparison. Finally, Rissanen et al^31^ performed an RCT of stepped-care stress management that is described as educationally based and uses CBT techniques. Their intervention compared group stress management and a similar individual approach, with a one-off education event used as a control. However, both interventions were not effective for cancer-related PTSD.

### EMDR

In their narrative review, Dimitrov et al^15^ also compared individual EMDR and CBT (delivered weekly for 8 weeks), with EMDR yielding the best outcomes. Participants had numerous cancer types (breast, colon, uterus, thyroid, melanoma, lung and stomach). Analysis showed that EMDR was equally effective in reducing PTSD symptoms in patients undergoing active treatment in addition to those in follow-up treatment. The authors concluded the study was at unclear risk of bias after assessment with a Cochrane quality tool, and the sample size was small, with participants predominantly being female and in their 50's. The review also included a study examining a group EMDR approach of six sessions over 3 days, but no control was mentioned in this study. Roberts^32^ performed case study research using group EMDR with 35 patients, comparing the treatment group with a delayed treatment group (1 month later). The results were unclear if the intervention was successful, but pre- and post-intervention scores appeared significantly better and were maintained at 90 days.

### Other therapeutic modalities

A broad range of other psychological approaches in cancer-related PTSD have been tried. Dimitrov et al^15^ examined telephone counselling with a resource booklet (16 sessions delivered over 12 months) against the resource booklet alone, with counselling yielding the best outcomes. Complimentary therapy (delivered twice weekly for 12 months) was also compared with a standard support group (delivered weekly for 12 weeks), with the support group showing the best outcomes. Monti et al^34^ performed case study research with seven patients, using a novel approach they termed the ‘neuro-emotional technique’ delivered over three sessions, which seems to be a blend of behavioural and cognitive techniques. ‘Short-term relief' of symptoms was noted as an outcome.

Offidani et al^37^ examined a 4-week programme of meditation aimed at improving attention and awareness while building essential skills to deal with stress. A control group was not included. The findings were unclear, but the authors suggested that patients with chronic stress report greater improvement in IES scores than those without stress symptoms using self-meditation techniques. There were specific improvements in the intrusive thoughts score and avoidance score that were statistically significant. Ochoa et al^38^ also used a so-called positive psychotherapy technique (12 weekly sessions of 90–120 min duration). The sessions focused on the assimilation of the cancer experience and encouraged personal transformation and psychological growth from the illness experience, using a manualised programme and guide. 126 patients were allocated to the experimental treatment, with patients also allocated to a waiting list control for 3 months. The intervention appeared to promote post-traumatic growth and reduced post-traumatic stress, with the benefits maintained at the 3- and 12-month follow-up.

Sarizadeh et al^33^ trialled acceptance and commitment therapy over 8 weeks, delivered as weekly sessions. This trial included a patient group and a survivors’ group. The same two groups were used for controls, with controls offered four sessions of treatment after completing the study. Acceptance and commitment therapy was effective in reducing demoralisation and cancer-related trauma symptoms in patients with breast cancer and in survivors. Finally, Chu et al^39^ examined a group-based expressive writing intervention that relied on cognitive reappraisal techniques and emotional disclosure. Three types of writing groups were examined: ‘cancer-fact’ (objective writing about cancer experiences), self-regulation and enhanced self-regulation. Significant differences in PTSD symptoms for all groups were noted. There were no significant differences in follow-up at 1, 3 and 6 months.

## Discussion

Group cognitive emotional work appears useful when compared with treatment as usual, and group CBT is also helpful in reducing cancer-specific thought intrusions. EMDR appears useful when compared with treatment as usual. When compared with individual CBT, EMDR appears to perform better. CBT appears to be more effective than counselling, which is more useful than a support booklet. Expressive writing intervention is also useful for hyperarousal and re-experiencing symptoms. Acceptance and commitment therapy is effective in reducing demoralisation and cancer-related trauma. Supportive care (including a one-off clinical appointment and telephone support over 2 months) is effective in reducing post-traumatic stress symptoms compared with controls. Positive psychotherapy intervention, focused on personal transformation and assimilation of cancer experience, promotes post-traumatic growth and reduces post-traumatic stress; the improvements were maintained at 1-year follow-up. It is unclear if meditation intervention worked for PTSD, although the authors did report that patients with chronic stress reported improvements as a result of the intervention.

### Implications

No studies in this review mentioned the use of patient and public involvement (PPI) in their study design. PPI is a growing field, and tools like the Involvement Matrix lay out processes to engage patients in study design, from preparation through to implementation.^40^ This review highlights the need to integrate PPI into study development, to ensure that patient and public views are captured and integrated into PTSD and cancer research. In addition to lack of PPI, this review highlights a lack of high-quality screening or robust interventions for cancer-related PTSD. A variety of approaches are used in the studies, including desensitisation and complimentary therapies, EMDR, expressive writing interventions and CBT-based techniques, some of which show promising results.

The heterogenous psychological approaches identified in this review contrast starkly to established guidelines for treating PTSD in the general population (as opposed to the cancer population). For example, in the UK, National Institute for Health and Care Excellence guidelines clearly outline a stepped approach to PTSD, using cognitive-based or EMDR approaches in the first instance.^41^ This narrative review also found that EMDR- and cognitive-based interventions were effective, alongside other approaches, and developing interventional RCTs to test out these approaches would be a useful next step. Although the UK clearly outlines treatment approaches to managing PTSD, there is a lack of robust policy specific to managing cancer-related PTSD; the 2022 10-year plan for cancer is delayed,^42^ and the latest National Health Service cancer programme report from 2020 is non-specific regarding meeting the emotional needs of people living with cancer.^43^ This is in contrast to the USA, where the National Cancer Institute advocates for screening and treatment of cancer-related PTSD.^44^ Furthermore, although not specific to cancer-related PTSD, development and rollout of a bespoke distress-screening tool in Toronto, Canada, led to a screening uptake of over 70% across the oncology department, suggesting that there is an appetite for screening and successful implementation of distress-screening tools, which could ultimately include cancer-related PTSD.^45^

In addition to screening, there is a clear identified need for cancer-related PTSD treatment that is bespoke to the cancer type and individual needs. Diagnostic clarity is important in the first instance, and this review shows a variety of diagnostic tools employed across the studies. Standardising the approach to diagnosis (e.g. by using DSM-5 criteria) would help to robustly identify affected patient populations and treatment needs. Furthermore, ongoing research is needed to develop an approach to identifying *de novo* cancer-related PTSD as opposed to pre-existing PTSD that has been triggered by a cancer-related experience. In addition, developing and testing bespoke interventions tailored to cancer type and patient population would be useful.

### Study and clinical limitations

There are limitations to this narrative review. Because of resource limitations, only English language papers were included, which could cause information bias as published research in other languages is missed from the review. Publication bias was not assessed because of the relatively small number of studies. Furthermore, there is known heterogeneity of PTSD diagnosis in included patient groups (pre-cancer diagnosis PTSD versus cancer-related PTSD). Definitions of ‘trauma’ and ‘distress’ vary in the literature, and, by restricting this review solely to patients with cancer with diagnosable PTSD, other studies that address fear of recurrence and post-traumatic stress symptoms have been omitted but may be relevant and applicable to the patient experience.^46^ There is also considerable heterogeneity in study participants, cancer types and quality of studies included, meaning it is difficult to draw firm conclusions about psychological interventions in PTSD in people living with cancer.

This review also noted low methodological quality in the studies included. RCTs typically failed to disclose the randomisation method used and the allocation concealment. It was unclear if the participants were subsequently informed if they were having the intervention or the control. Few studies described the drop-out rates and factors behind participants dropping out. No study mentioned intention-to-treat analysis as a management strategy and few studies calculated effect sizes.

In conclusion, PTSD in cancer is common, but there is a lack of high-quality intervention studies for PTSD in cancer. A need for diagnostic clarity and associated intervention RCTs has been identified. Additionally, a need for interventional RCTs for managing PTSD in people living with cancer has also been identified. Diagnostic clarity, using updated DSM-5 criteria for PTSD, and a consistency of patient population in terms of demographics and cancer type and stage, may enable sufficient homogeneity of studies to permit a meta-analysis. However, given the diversity of PTSD and cancer needs identified, this is unlikely to be achievable. In this regard, bespoke and specific studies that tailor the PTSD intervention to particular cancer populations under investigation is a pragmatic argument based on psychological formulation that will permit meaningful real-world data to be generated. In a similar vein, research that differentiates *de novo* cancer-related PTSD from patients with cancer with pre-existing PTSD is required.

Consequently, it is the authors’ intentions that this narrative review, alongside forthcoming interviews of people living with cancer and their experiences of PTSD, will inform a novel intervention to screen, diagnose and manage cancer-related PTSD. No studies to date have mentioned use of PPI in study design, and there is seemingly a lack of patient and public engagement in the designing of effectiveness studies in current PTSD and cancer research, which is a concern. Furthermore, there is a lack of robust UK policy specific to managing cancer-related PTSD, which differs from the National Institutes of Health in the USA, which advocates for screening and treatment of cancer-related PTSD.^44^ It is hoped the narrative reviews to date, alongside this review and PPI, will generate better-designed trials to inform local policy.

## Data Availability

Data is available on reasonable request from the corresponding author.

## About the authors

**Daniel Anderson** is a consultant psychiatrist at the Department of Psych-oncology, The Christie NHS Foundation Trust, Manchester, UK. **Victoria Jones** is a specialty trainee (ST4) psychiatrist in adult psychiatry, Greater Manchester Mental Health NHS Foundation Trust, Manchester, UK.
